# Inducible Expression of *spo0A* as a Universal Tool for Studying Sporulation in *Clostridium difficile*

**DOI:** 10.3389/fmicb.2017.01793

**Published:** 2017-09-21

**Authors:** Marcin Dembek, Stephanie E. Willing, Huynh A. Hong, Siamand Hosseini, Paula S. Salgado, Simon M. Cutting

**Affiliations:** ^1^Institute for Cell and Molecular Biosciences, Newcastle University Newcastle upon Tyne, United Kingdom; ^2^School of Biological Sciences, Royal Holloway, University of London London, United Kingdom

**Keywords:** sporulation, *Clostridium difficile*, *spo0A*, spore, inducible expression, allele-coupled exchange, anhydrotetracycline

## Abstract

*Clostridium difficile* remains a leading nosocomial pathogen, putting considerable strain on the healthcare system. The ability to form endospores, highly resistant to environmental insults, is key to its persistence and transmission. However, important differences exist between the sporulation pathways of *C. difficile* and the model Gram-positive organism *Bacillus subtilis*. Amongst the challenges in studying sporulation in *C. difficile* is the relatively poor levels of sporulation and high heterogeneity in the sporulation process. To overcome these limitations we placed *P_tet_* regulatory elements upstream of the master regulator of sporulation, *spo0A*, generating a new strain that can be artificially induced to sporulate by addition of anhydrotetracycline (ATc). We demonstrate that this strain is asporogenous in the absence of ATc, and that ATc can be used to drive faster and more efficient sporulation. Induction of Spo0A is titratable and this can be used in the study of the *spo0A* regulon both *in vitro* and *in vivo*, as demonstrated using a mouse model of *C. difficile* infection (CDI). Insights into differences between the sporulation pathways in *B. subtilis* and *C. difficile* gained by study of the inducible strain are discussed, further highlighting the universal interest of this tool. The *P_tet_-spo0A* strain provides a useful background in which to generate mutations in genes involved in sporulation, therefore providing an exciting new tool to unravel key aspects of sporulation in *C. difficile.*

## Introduction

*Clostridium difficile* is a Gram-positive, anaerobic, spore-forming pathogen and the leading cause of healthcare associated diarrhea worldwide (Rupnik et al., 2009; Smits et al., 2016). *C. difficile* infection (CDI) occurs most commonly in nosocomial settings, among patients whose natural gut microbiota has been perturbed by prolonged treatment with broad-spectrum antibiotics (Dethlefsen et al., 2008; Lawley and Walker, 2013). Two large cytotoxins secreted by the vegetative cell are responsible for the clinical manifestations of CDI, which range from mild, self-limiting diarrhea to severe, often fatal inflammatory complications, such as pseudomembranous colitis or toxic megacolon (Dubberke and Olsen, 2012). However, it is the spore that is the primary infectious agent, as mutant strains defective in sporulation are unable to efficiently persist in the environment and transmit disease (Deakin et al., 2012). Due to their multi-layered structure, spores are extremely robust and resistant to both chemical and physical insults, enabling *C. difficile* to survive exposure to heat, oxygen, alcohol, noxious chemicals, and certain disinfectants (Lawley et al., 2010; Vohra and Poxton, 2011). Despite its key relevance in CDI, many aspects of sporulation in *C. difficile* are still understudied, hampered by low sporulation efficiency and high heterogeneity in the sporulation process.

In order to sporulate, the cell must undergo an ordered program of unicellular differentiation that leads to a dormant, non-replicative state. The decision to enter the developmental program is highly regulated, requiring multiple environmental and metabolic cues (Al-Hinai et al., 2015). It is mediated by Spo0A, a conserved transcription factor that plays a central role in phenotypic differentiation and adaptation to changing environments. The role of Spo0A in orchestrating sporulation has been studied in exquisite detail in *Bacillus subtilis* (reviewed in Sonenshein, 2000). Spo0A is post-translationally activated *via* phosphorylation facilitated by a complex phosphorelay consisting of several sensor kinases (KinA-E). These kinases respond to external signals by auto-phosphorylating and subsequently transferring a phosphate residue either directly to Spo0A or *via* intermediary proteins Spo0F and Spo0B (Jiang et al., 2000b). In contrast to *B. subtilis*, there are no Spo0F or Spo0B homologs, and therefore presumably no phosphorelay system, in *C. difficile;* rather, Spo0A is believed to be phosphorylated directly by the sensor kinases (Underwood et al., 2009; Steiner et al., 2011).

The phosphorelay affords opportunity for multiple layers of regulation, such as Rap phosphatases that modulate the level of phosphorylated Spo0A, permitting a degree of fine-tuning and flexibility to the system (Veening et al., 2005). It remains unclear whether this level of control is absent in *C. difficile* or is achieved by other means (Jiang et al., 2000a; Paredes et al., 2005). Studies published to date have shown that *C. difficile* initiates sporulation in response to nutrient availability (Edwards et al., 2014; Nawrocki et al., 2016) and that a Rap-like protein, RtsA, acts as a positive regulator of sporulation, although the exact nature of this effect remains unclear (Edwards et al., 2016). Sporulation, initiated by an asymmetric division of the vegetative cell into a smaller forespore and a larger mother cell, then proceeds *via* an expression cascade controlled by sporulation-specific sigma factors (σ^E^, σ^F^, σ^G^, and σ^K^). Differential expression of sigma factors in each of those compartments allows the sporulation process to be completed.

Recent studies of Spo0A in *B. subtilis* have demonstrated that the importance of this regulator extends beyond just sporulation, revealing the existence of an intricate Spo0A regulatory network fine-tuned to respond to the specific amount of Spo0A present in the cell (Fujita et al., 2005; Vishnoi et al., 2013). Efficient sporulation requires a progressive increase in the levels of Spo0A, which prompts the gradual activation initially of low-threshold Spo0A-regulated genes and subsequently high-threshold Spo0A-regulated genes (Fujita et al., 2005; Vishnoi et al., 2013). In *B. subtilis* the Spo0A regulon comprises approximately 120 genes (Molle et al., 2003). At low levels of Spo0A, genes involved in lifestyle changes that might delay the need to sporulate, such as biofilm formation, are activated and only if levels of Spo0A continue to rise until a threshold level is reached does the cell commit to sporulation (Fujita et al., 2005). Thus, Spo0A serves as a focal point for the integration of a number of factors that determine whether the cell needs to enter a dormant state. In *C. difficile*, Spo0A is thought to control approximately 300 genes (Fimlaid et al., 2013; Pettit et al., 2014), many of which have been linked to biofilm formation (Dawson et al., 2011), swimming motility (Pettit et al., 2014) and toxin production (Underwood et al., 2009), although conflicting results have been reported across different strains and laboratories (Rosenbusch et al., 2012; Mackin et al., 2013), indicating the complex nature of Spo0A signaling.

In an attempt to provide a more robust and tractable way to study sporulation in *C. difficile*, we have generated an inducible *spo0A* strain and investigated whether controlled expression of the transcription factor is a viable method for achieving higher sporulation efficiency and homogeneity. We demonstrate that *spo0A* expression can be induced in a dose-dependent manner and that the level of expression correlates with sporulation efficiency, which reaches values higher than those observed in an isogenic wild type (WT) strain. We also show that *spo0A*-dependent genes involved in other aspects of the bacterial life cycle can be indirectly controlled by use of the inducing agent. Finally, using the mouse model of CDI, we demonstrate that induction of *spo0A* can be carried out *in vivo*, providing a new tool in studying the role of Spo0A-dependent phenotypes in virulence.

## Results

### Generating an Inducible *spo0A* Strain

**FIGURE 1:**
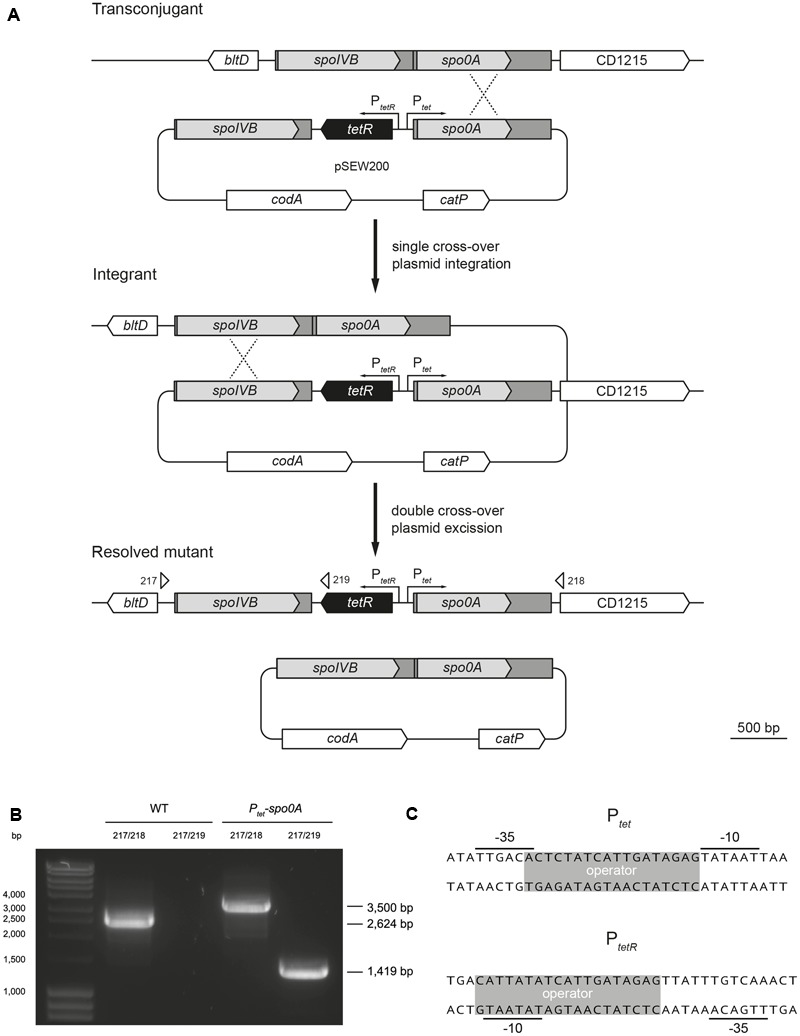
Confirmation of *P_tet_* integration into the *C. difficile* 630 chromosome. **(A)** Stepwise diagram detailing the stages of ACE-mediated mutagenesis. Upstream and downstream homology regions spanning the *spoIVB* and *spo0A* ORFs are indicated as gray boxes. The P*_tet_* regulatory elements are depicted in black. Binding sites for primers used in PCR screening are indicated with arrows. **(B)** Agarose gel electrophoresis analysis of PCR products obtained during screening for WT and P*_tet_*-*spo0*A. Expected products sizes are indicated on the right side of the panel. **(C)** Detailed representation of the *P_tet_* regulatory elements inserted upstream of *spo0A* (CD1214).

Allele-coupled exchange was used to place the inducible *P_tet_* promoter with its regulatory elements 30 bp upstream of the *spo0A* ORF on the *C. difficile* 630 chromosome, generating *P_tet_-spo0A* (**Figure 1A**). PCR and sequencing of the resulting product was used to confirm correct integration of the promoter (**Figure 1B**). The *tet* element comprises two divergent promoters, *P_tet_* and *P_tetR_*, each overlapping with a *tet* operator sequence and regulated by tetracycline (**Figure 1C**). TetR, which represses both *P_tet_* and *P_tetR_*, introduces a negative feedback loop to the system that is tightly dose-dependent, allowing an increased level of control (Fagan and Fairweather, 2011). To minimize potential confounding factors resulting from using an antibiotic, the non-antibiotic tetracycline analog anhydrotetracycline (ATc) was used as the inducing agent. To exclude the possibility that ATc was exerting a deleterious effect on normal growth, WT and *P_tet_-spo0A* cultures were incubated in the presence or absence of ATc and their growth rate was monitored over time by measuring optical density (OD_600_). No appreciable difference in growth rate was observed between WT and *P_tet_-spo0A*, irrespective of the ATc concentration used (Supplementary Figure S1).

### Expression of *spo0A* and *spo0A*-Dependent Genes in *P_tet_-spo0A* Can be Controlled in Response to ATc

**FIGURE 2:**
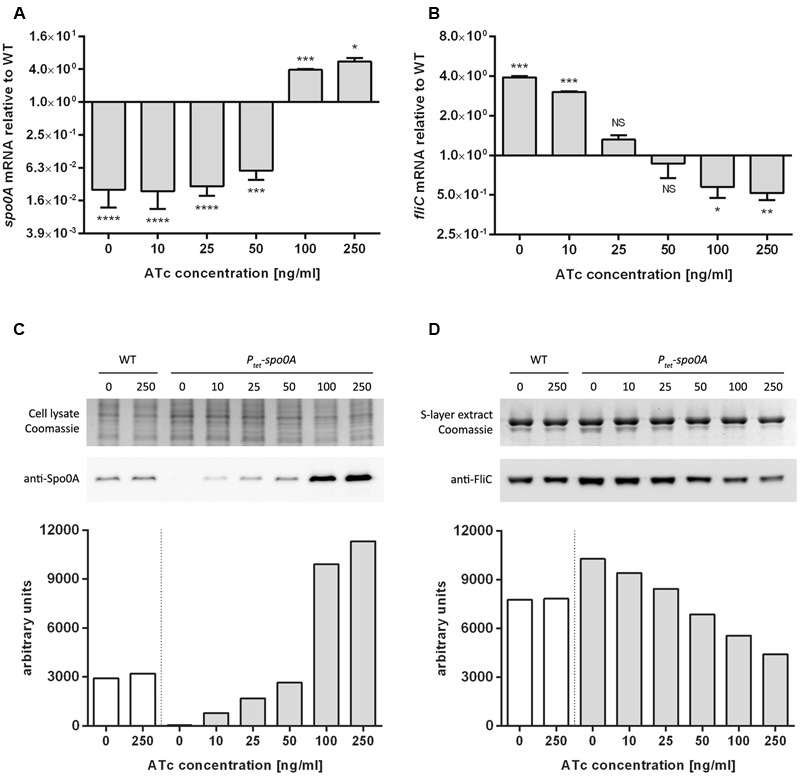
Analysis of dose-dependent expression of *spo0A* and *fliC* in *P_tet_-spo0A.* qRT-PCR analysis of *spo0A* **(A)** and *fliC* **(B)** transcripts isolated from WT or *P_tet-_spo0A* grown with increasing concentrations of ATc. Data displayed is *P_tet-_spo0A* relative to WT (^∗^*p* ≤ 0.05; ^∗∗^*p* ≤ 0.01; ^∗∗∗^*p ≤* 0.001; ^∗∗∗∗^*p ≤* 0.0001 by a two-tailed Student’s *t*-test). Densitometric analysis of anti-Spo0A **(C)** and anti-FliC **(D)** immunoblots on protein extracts form WT or *P_tet-_spo0A* cells grown with increasing concentrations of ATc. Coomassie-stained gels are provided as loading control.

To verify whether *spo0A* expression can be driven by the introduced *P_tet_* promoter upon induction, qRT-PCR was used to measure the level of *spo0A* transcripts in *P_tet_*-*spo0A* relative to WT following growth with a range of ATc concentrations spanning 0–250 ng/ml. A clear dose-dependent response in transcript levels was observed (**Figure 2A**). In the absence of the inducing agent, *P_tet_-spo0A* showed an approximately 40-fold reduction in detectable *spo0A* mRNA when compared to WT (*p* < 0.0001), a background level of expression possibly attributable to a small amount of promoter ‘leakage.’ Transcript levels gradually increased with rising ATc concentration, with the largest change observed between 50 and 100 ng/ml, where the levels of *spo0A* transcripts in *P_tet_*-*spo0A* were fourfold higher than those found in WT at 100 ng/ml. At the highest concentration of ATc used, 250 ng/ml, *spo0A* transcript levels were approximately 5.6-fold higher in the mutant relative to WT grown at the same concentration of ATc (*p* = 0.0161) (**Figure 2A**).

Spo0A is linked to many phenotypes in *C. difficile*. One of these is flagella-mediated motility where Spo0A has been shown to negatively regulate a number of genes, including *fliC* (Pettit et al., 2014). To further validate the *P_tet_-spo0A* system and to demonstrate that the strain can be used to study *spo0A*-related phenotypes beyond sporulation, we sought to confirm the link between *fliC* and *spo0A* by way of ATc induction. In keeping with previous reports on the link between Spo0A and FliC, qRT-PCR demonstrated that *fliC* transcription levels decreased with increasing ATc concentrations, dropping from 3.9-fold (*p* = 0.0003) to 0.5-fold (*p* = 0.0075) relative to WT (**Figure 2B**). To confirm that the dose-dependent changes in *spo0A* and *fliC* transcript levels observed upon ATc induction correlated with similar changes in protein levels, OD-normalized protein extracts from *P_tet_-spo0A* cultures grown with a range of ATc concentrations were probed for Spo0A and FliC by Western blotting. In line with our qRT-PCR results, levels of Spo0A in whole cell lysates increased with the increase in ATc concentration while the amount of FliC in surface layer (S-layer) extracts decreased. Importantly, the amount of Spo0A and FliC remained constant in WT, irrespective of ATc concentration (**Figures 2C,D**).

### Sporulation Efficiency in *P_tet_-spo0A* Can be Controlled in Response to ATc

**FIGURE 3:**
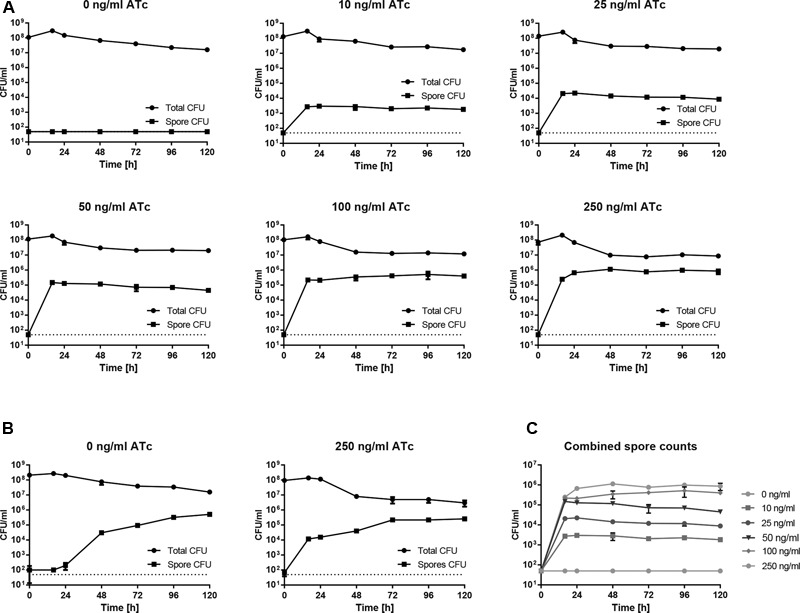
Dose-dependent increase in sporulation efficiency in *P_tet_-spo0A. P_tet_-spo0A* **(A)** or WT **(B)** were grown to OD ∼0.8 and sub-cultured 1:10,000 in fresh BHIS supplemented with the concentration of ATc indicated, or 1% ethanol (0 ng/ml ATc). Samples were removed at the indicated time points post-inoculation, serially diluted in sterile, pre-reduced PBS and plated onto BHIS agar supplemented with 0.1% Tch with (spore CFU) or without (total CFU) prior heat-treatment at 70°C for 30 min. Colonies were enumerated after 24 h of incubation under anaerobic conditions. **(C)** Combined *P_tet_-spo0A* counts showing a dose dependent increase in sporulation efficiency. Data presented as means ± SD from three technical replicates. Each result is representative of experiments performed in at least biological triplicate. Dotted line represents the limit of detection.

Studies reported to date have shown substantial variation in sporulation between different strains of *C. difficile* (Underwood et al., 2009; Burns et al., 2010; Vohra and Poxton, 2011). Accordingly, making comparisons between strains has proven problematic and is further compounded by a lack of uniform methods for induction and assessment of sporulation (reviewed in Burns and Minton, 2011). Irrespective of the methodologies used, efficiency of sporulation under laboratory conditions appears to be low, particularly when compared to *B. subtilis*, hampering work on the molecular basis of sporulation in *C. difficile*. Having shown that *spo0A* expression can be manipulated in *P_tet_-spo0A via* induction with ATc, we asked whether the observed changes in Spo0A levels would affect the overall sporulation efficiency. We first determined whether the *tet* element is sufficiently tightly regulated to render the *P_tet_-spo0A* strain asporogenous in the absence of ATc. Supporting this, after 120 h of nutrient starvation in liquid culture, typically regarded as the time after which the sporulation process of a *C. difficile* culture is complete (Burns and Minton, 2011), no spores could be detected based on heat-resistant CFU counts (**Figure 3B**). As expected, *P_tet_-spo0A* cultures grown in the presence of increasing concentrations of ATc showed a dose-dependent increase in heat-resistant CFUs within 16 h from induction. Maximum spore titers were observed at 24–48 h post-inoculation, reaching 3 × 10^3^ ± 3.28 × 10^2^ CFU/ml; 2.25 × 10^4^ ± 1.32 × 10^3^ CFU/ml; 1.48 × 10^5^ ± 2.47 × 10^4^ CFU/ml; 5.17 × 10^5^ ± 2.75 × 10^5^ CFU/ml and 9.83 × 10^5^ ± 1.04 × 10^5^ CFU/ml in cultures induced with 10, 25, 50, 100, and 250 ng/ml of ATc respectively (**Figures 3A,C**), and remained largely constant throughout the duration of the experiment. Taking into account the total CFU observed at t0, these values correspond to a sporulation efficiency of 0.001% (10 ng/ml ATc), 0.006% (25 ng/ml ATc), 0.039% (50 ng/ml ATc), 0.387% (100 ng/ml ATc), and 1.209% (250 ng/ml ATc) showing a clear dose response. Importantly the observed titers were reached faster and, at the maximum concentration of ATc, were significantly higher than in WT (2.58 × 10^5^ ± 1.25 × 10^4^ CFU/ml) (*p =* 0.0364) (**Figure 3B**). While the inducing agent had no effect on the final spore titers observed in WT cultures, it did change the dynamics of the sporulation process, resulting in higher spore titers at 16–24 h post-induction (**Figure 3B**), possibly an effect of additional stress that high concentration of ATc exerts on the cells.

### Controlled Expression of *spo0A* Has No Effect on Overall Homogeneity of Sporulating Cultures

**FIGURE 4:**
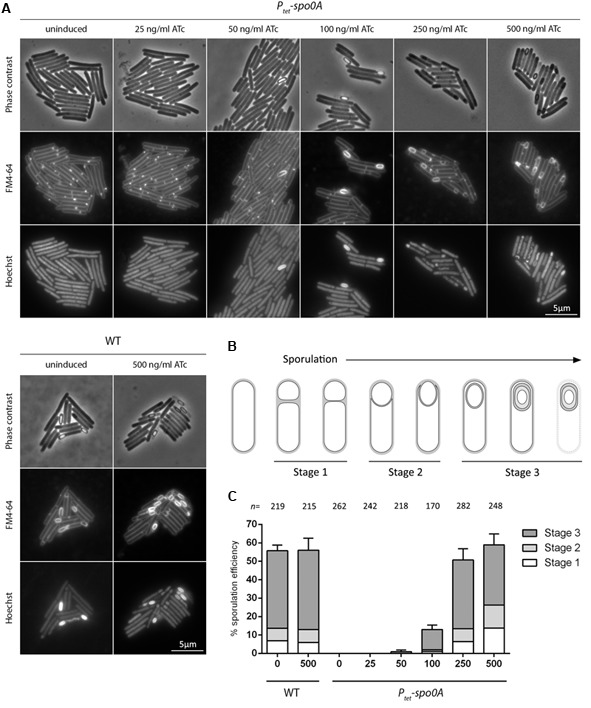
Analysis of *P_tet_*-*spo0A* sporulation dynamics. **(A)** WT and *P_tet_-spo0A* cultures were harvested after 14 h of growth on 70:30 agar supplemented with ATc as indicated. Cells were stained with FM4-64 and Hoechst 33258 and analyzed by phase contrast and fluorescence microscopy. Scale bar corresponds to 5 μm. **(B)** Schematic representation of the sporulation process indicating stages corresponding to asymmetric cell division (stage 1), engulfment (stage 2) and spore maturation (stage 3). **(C)** Sporulation efficiency and distribution of cells at different stages of sporulation in WT and *P_tet_-spo0A* grown for 14 h on 70:30 agar supplemented with ATc as indicated. Sporulation efficiency is expressed as the percentage ratio of cells at a given stage of sporulation to total cells counted. Data reported as means ± SD from 10 random fields of view. The total number of cells counted (n) is indicated above each column on the graph.

The lack of synchronicity observed in sporulating *C. difficile* cultures has been a significant barrier to population-wide studies as any measurements have to be interpreted as an average value across cells at different stages of the bacterial life cycle. Since sporulation in *P_tet_-spo0A* under ATc induction was capable of proceeding faster than in WT, we asked whether this would be reflected in a more synchronous process. To investigate this, we monitored sporulating *P_tet_-spo0A* cultures grown in the presence of increasing ATc concentrations by fluorescence microscopy following staining with FM4-64 and Hoechst 33258 to visualize the membrane and DNA, respectively. In order to overcome the high limit of detection of the technique, cultures were sporulated on a 70:30 mixture of sporulation media (SM) and BHIS agar, previously used in sporulation studies by several groups as it seems to provide faster and more efficient sporulation (Fimlaid et al., 2013; Putnam et al., 2013; Edwards et al., 2016). In line with our previous experiments conducted in liquid culture, a clear dose response in sporulation efficiency was observed, although a twofold higher concentration of ATc had to be used to reach saturation, presumably due to limited ATc availability in plated culture when compared to liquid culture. Mean values of sporulation efficiency, calculated as the percentage ratio of cells that had undergone asymmetric cell division to total cells counted, reached 0.47 ± 1%; 12.37 ± 2.35%; 50.44 ± 6.17%, and 58.99 ± 6.02% in cultures induced with 50, 100, 250, and 500 ng/ml ATc, respectively. No sporulation was observed in cultures induced with 25 ng/ml ATc or in uninduced cultures. As expected, ATc had no significant effect on sporulation efficiency in WT (**Figure 4A**), reaching 55.6 ± 3.04% in the uninduced culture and 55.93 ± 6.59% in cultures induced with 500 ng/ml ATc. In order to assess whether controlled expression of *spo0A* has an effect on the overall homogeneity of sporulation, we examined the composition of sporulating cultures by counting cells that had reached one of three defined morphological stages: (i) cells with flat, asymmetric septa; (ii) cells undergoing engulfment and (iii) cells with fully engulfed sporangia (including mature spores) (**Figure 4B**). While the distribution of cells at stage 1, 2, or 3 did change with increasing ATc concentration, the overall homogeneity of the culture did not improve when compared to WT, particularly at higher concentrations of ATc required to match WT sporulation efficiency, suggesting that controlled expression of *spo0A* on its own does not increase the synchronicity of the sporulating culture (**Figures 4A,C**).

### Use of *P_tet_-spo0A* as a Background Strain in Studies of Sporulation-Related Phenotypes

**FIGURE 5:**
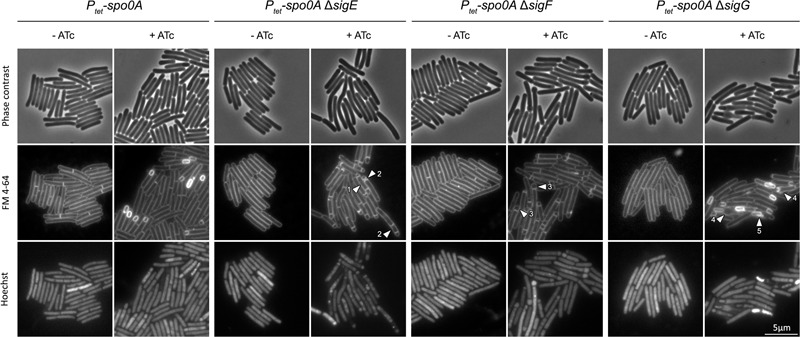
Morphological characterisation of *P_tet_-spo0A*Δ*sigE, P_tet_-spo0A* Δ*sigF*, and *P_tet_*-*spo0A* Δ*sigG* mutants. Sporulating cultures of *sigE, sigF*, and *sigG* mutants created in the *P_tet_-spo0A* background were harvested after 14 h of growth on 70:30 agar supplemented with ATc as indicated. Cells were stained with FM4-64 and Hoechst 33258 and analyzed by phase contrast and fluorescence microscopy. Arrows indicate: (1) abortive diasporic forms, (2) multiple polar septa, (3) forespore bulging, (4) mother cell bulging and (5) dislodged sporangia resting at an angle to the longitudinal axis of the cell. Scale bar corresponds to 5 μm.

One of the main benefits of using allele-coupled exchange to genetically engineer *C. difficile* is the possibility of creating strains carrying multiple mutations through subsequent rounds of mutagenesis since any changes to the chromosome are left unmarked and therefore not reliant on the limited availability of suitable antibiotic resistance markers (Cartman et al., 2012; Heap et al., 2012). Having shown that inducible expression of *spo0A* is a viable strategy for controlling sporulation in *C. difficile*, we set out to investigate whether *P_tet_-spo0A* could be used as a parental strain in studies of sporulation-related genes. As ‘proof of concept’ we have created *sigE, sigF*, and *sigG* mutants in the *P_tet_-spo0A* background by introducing in-frame deletions into the respective genes by allele-coupled exchange. The resulting strains designated *P_tet_-spo0A*Δ*sigE, P_tet_-spo0A*Δ*sigF*, and *P_tet_-spo0A*Δ*sigG* were sporulated on solid media in the presence or absence of ATc and analyzed by microscopy following membrane and DNA staining (**Figure 5**). When induced, both the *sigE* and *sigF* mutants were blocked at the asymmetric division stage, revealing abortive disporic cells and occasionally multiple, closely located polar septa. Given the apparent lack of DNA staining in compartments created by these multiple septa, the observed staining pattern could equally be the result of membrane delamination between the forespore and the mother cell. Additionally, *sigF* mutant forespores showed distinct bulging. While cells of the *sigG* mutant were able to complete engulfment, they did not proceed further in morphogenesis and the resulting forespores were often misshapen and dislodged, resting at an angle to the longitudinal axis of the cell. As expected, strains grown in the absence of ATc did not initiate sporulation (**Figure 5**). These observations are largely consistent with previous work characterizing the morphology of sporulation-specific sigma factor mutants created using ClosTron insertional mutagenesis (Pereira et al., 2013) and demonstrate the potential of using *P_tet_-spo0A* in studies that would benefit from more control over the sporulation process.

### Use of *P_tet_-spo0A* in the Mouse Model of Infection

The *spo0A* regulatory network co-ordinates multiple aspects of the *C. difficile* life cycle, including toxin production and the resulting virulence of a given strain. Deakin et al. (2012) reported that a R20291 Δ*spo0A* strain (ribotype 027) produced more toxin *in vitro* and caused more severe disease than WT in a murine model of infection, while being deficient in persistence and transmission. More recent studies however, suggest that this effect is not consistent across all *C. difficile* strains (Mackin et al., 2013). While Spo0A was confirmed to be a negative regulator of toxin production in ribotype 027 strains, no such effect was observed in ribotype 012, suggesting that Spo0A could differentially regulate toxin production between phylogenetically distinct strains of *C. difficile* (Mackin et al., 2013). The exact mechanisms of the increased virulence of some *spo0A* mutants are not yet known, but the explanation is most likely to be multi-faceted. In *B. subtilis*, Spo0A does not have an equal affinity for all genes within the *spo0A* regulon, leading to a programmed change in gene expression with sporulation-specific genes not activated until high levels of Spo0A have accumulated (Fujita et al., 2005). An inducible system that offers titratable control over Spo0A levels provides an opportunity to ask more nuanced questions about the role of Spo0A in the infectious process in a manner that the Δ*spo0A* mutant does not. Previous studies have demonstrated the potential for ATc induction of bacterial gene expression in mouse models (Ji et al., 2001; Kotula et al., 2014). Therefore, having characterized the *P_tet_-spo0A* strain *in vitro*, we asked whether the inducible system would also work *in vivo*.

**FIGURE 6:**
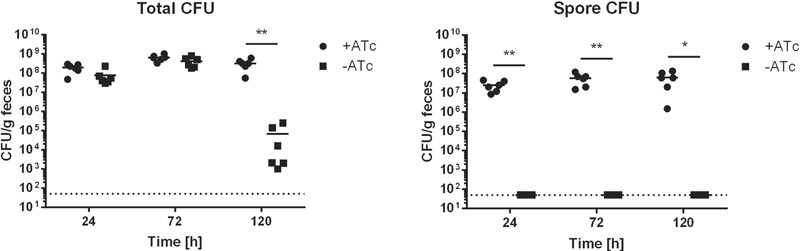
Use of the *P_tet_* inducible system *in vivo.* 12 mice were treated with clindamycin to disrupt the natural flora. All mice were subsequently infected with the same culture of *P_tet_*-*spo0A* grown overnight without ATc (infectious dose = 1 × 10^7^ CFU). The mice were then split into two groups of 6, one of which received 1% ethanol in their drinking water, and the other 100 μg/ml ATc. Feces were collected at the time indicated post-infection and enumerated for total and heat-resistant CFUs. Data plotted for each animal individually with mean indicated (^∗^*p* ≤ 0.05; ^∗∗^*p* ≤ 0.01 by a multiple Student’s *t*-test using the Holm-Sidak method). Dotted line represents the limit of detection.

In our study, 12 mice pre-treated with clindamycin were infected with 1 × 10^7^ CFU from an overnight culture of *P_tet_-spo0A* grown without ATc. Six mice received 0.1 mg/ml ATc in their drinking water, with the remaining six receiving 1% (v/v) ethanol as a negative control. Feces were collected at 24, 72, and 120 h post-infection, and total and heat-resistant CFUs measured (**Figure 6**). No statistically significant difference was found for total cell counts between the ATc^-^ and ATc^+^ groups until 120 h after infection, indicating successful infection of both groups. For the group of mice that did not receive ATc in their drinking water, no spores could be detected by enumeration of heat resistant colonies, indicating a failure to sporulate within the mouse. In contrast, for the group of mice that received 0.1 mg/ml ATc in their drinking water, heat-resistant CFUs were detected 24 h post-infection, steadily increasing until the final time-point taken 120 h post-infection. To confirm the identity of the isolated strains post-infection, five colonies from each group were selected at random and screened for *P_tet_-spo0A* by PCR. As shown in Supplementary Figure S2, all strains were found to carry *P_tet_-spo0A*. Thus, the *P_tet_* promoter does not become activated *in vivo*, rendering *P_tet_-spo0A* asporogenous. Furthermore, ATc can be used to control the *P_tet_* system *in vivo.* Therefore, this approach will permit the study of the role of Spo0A and sporulation/germination kinetics in infection with a level of control not possible with previously used systems.

## Discussion

Sporulation is a key contributing factor in establishing *C. difficile* as a leading nosocomial pathogen in the developed world. Studies carried out in *B. subtilis* have undoubtedly provided a useful basis for study of sporulation in Clostridial species, but it has become clear that there are several key differences in the sporulation process which make extrapolating results difficult. These differences are most apparent at the stage of entry into sporulation, governed in *Bacilli* by a phosphorelay system that is absent in Clostridial species. It is presumed that the phosphorelay system provides tighter regulation over the commitment to sporulate, but this premise remains unproven and the consequences of the lack of such a system remain unclear. Another aspect of the *C. difficile* life cycle that is uniquely affected by Spo0A is toxin production, a phenotype that cannot be studied in a non-toxigenic species such as *B. subtilis*. Additionally, Spo0A regulates other lifestyle changes in a manner that can vary from species to species and even between strains. Thus, although Spo0A is the master regulator of sporulation, it is also an important instigator of transcriptional changes that might help evade the need to sporulate, such as cannibalism or biofilm formation.

Under laboratory conditions, *C. difficile* sporulation efficiency is low compared to *B. subtilis* (Burns and Minton, 2011), a technical hurdle to its study that is exacerbated by the high heterogeneity and the current inability to synchronize a sporulating culture. Furthermore, as illustrated in studies of *B. subtilis*, the study of *spo0A-*null mutants does not permit recognition of the importance of incremental increases in the level of Spo0A, leading to more nuanced Spo0A-mediated phenotypes to be overlooked. An over-simplification of the intricate Spo0A network is perhaps one reason for conflicting results regarding Spo0A-mediated phenotypes in the literature. In this study, we report the generation of an inducible *spo0A* system, as schematically detailed in **Figure 1A**, in an attempt to improve sporulation efficiency, investigate the possibility of achieving a more homogenous culture, and test *spo0A-*titratable phenotypes.

In WT *C. difficile* cultures, heat-resistant CFUs are typically observed 8–12 h after reaching stationary phase (Pereira et al., 2013) and the sporulation process can take up to 5 days to reach equilibrium (Burns and Minton, 2011). We have demonstrated that inducing *spo0A* expression significantly reduces the amount of time necessary to reach maximum spore titers. Analysis of the cultures by fluorescence microscopy indicated that sporulation proceeds similarly to WT even when induction leads to faster completion of sporulation, but transmission electron microscopy would be required to rule out any changes to the fine structure of the spore. These results demonstrates that induction of Spo0A is sufficient to drive sporulation and, at least to some degree, overrides the need for external stimulus. Therefore, the *P_tet_-spo0A* strain is a useful background for generating mutants in genes involved in other stages of the sporulation pathway, as illustrated by construction of sporulation-specific sigma factor mutants. Furthermore, the *P_tet_* promotor removes *spo0A* from its normal regulatory network; this allows the maintenance of high levels of *spo0A* expression when mutation of a gene in the sporulation network might otherwise serve to downregulate *spo0A* and potentially mask phenotypes of interest.

Our finding that accelerated accumulation of Spo0A correlates with more rapid sporulation contrasts with studies performed in *B. subtilis*, where accelerated accumulation is detrimental to sporulation (Vishnoi et al., 2013). We found no evidence that fast accumulation to elevated levels of Spo0A has any detrimental effect on sporulation, indicating that a gradual build-up of Spo0A is not important to the sporulation process itself in *C. difficile*. In *B. subtilis*, it has been suggested that the reduction in sporulation efficiency upon artificial induction of Spo0A resulted from premature repression of DivIVA, which is involved in chromosome segregation during the earlier stages of sporulation but downregulated *via* Spo0A in the later stages of sporulation (Vishnoi et al., 2013). Existing transcriptomic and proteomic studies of *C. difficile* Δ*spo0A* have not found a statistically significant regulation of DivIVA by Spo0A (Pettit et al., 2014; Charlton et al., 2015), but the *P_tet_-spo0A* strain will allow a more in-depth study of this connection, as well as investigations into the potential existence of high and low *spo0A* regulons more generally. It has been previously proposed that the phosphorelay system present in *B. subtilis* is responsible for the gradual build-up of Spo0A (Fujita and Losick, 2005) and our finding would be in line with this notion.

Finally, we demonstrated that the inducible system works in a mouse model of infection, with *P_tet_-spo0A* remaining asporogenous in the absence of ATc in drinking water but sporulating in its presence. Although beyond the scope of this work, *P_tet_-spo0A* could be used to investigate germination rates and efficiency *in vivo*, following infection with spores produced by ATc induction *in vitro* which will be unable to re-sporulate *in vivo*. Furthermore, we envisage that administration of ATc at different doses relative to mouse weight will allow dose-dependent phenotypes to be studied during infection. This paves the way for more detailed study into the role of Spo0A in virulence, and to delineate the relative importance of the many Spo0A related factors involved. The *P_tet_-spo0A* strain described here is therefore a novel tool to study sporulation in *C. difficile* in a much more robust and controllable way, that will allow us to gain unique insights into the mechanisms involved in spore formation and related phenotypes, as well as the role of Spo0A in pathways leading to different lifecycle choices.

## Materials and Methods

### Bacterial Strains and Growth Conditions

*Escherichia coli* strains were grown in LB-Miller supplemented where necessary with chloramphenicol (15 μg/ml) and/or kanamycin (50 μg/ml). *C. difficile* strains were cultured statically in BHIS (37 g brain heart infusion, 5 g yeast extract and 1 g L-cysteine per liter), SM (90 g Bacto peptone, 5 g proteose peptone, 1 g NH_4_SO_4_, 1.5 g Tris base) or a 70:30 mixture of SM and BHIS as indicated, supplemented where necessary with thiamphenicol (15 μg/ml), D-cycloserine (250 μg/ml), 5-fluorocytosine (FC) (50 μg/ml) or anhydrotetracycline (ATc) (5–500 ng/ml). For plate cultures, media were solidified with 1.5% (w/v) agar. All *C. difficile* strains were grown and maintained at 37°C in a Don Whitley DG250 anaerobic workstation under anaerobic conditions (10% H_2_, 10% CO_2_, 80% N_2_) (Don Whitley Scientific). A detailed list of strains used in this study is provided in Supplementary Table S1.

### Construction of *P_tet_-spo0A* Mutant

To generate plasmid pSEW200, the following DNA fragments were PCR-amplified using KOD Hot Start polymerase (Novagen). The pMTL-SC7315 plasmid backbone (Cartman et al., 2012) was linearized by inverse PCR using primers 103 and 104. 1,200 bp upstream and downstream *spo0A* homology arms were amplified from *C. difficile* 630 genomic DNA, using primer pairs 155–156 and 159–160, respectively. *Tet* regulatory elements were amplified from pRPF185 (Fagan and Fairweather, 2011) using primer pair 157–158. The resulting fragments were ligated using Gibson assembly (NEB) as per manufacturer’s instructions. pSEW200 was transformed into *E. coli* CA434 and then conjugated into either *C. difficile* 630 or *C. difficile* 630Δ*erm* as described previously (Purdy et al., 2002). Resulting colonies were restreaked twice onto fresh BHIS agar plates supplemented with thiamphenicol and D-cycloserine to counter-select for *E. coli.* Allelic exchange was performed as described previously (Cartman et al., 2012), with the slight modification of colonies being restreaked three times onto *C. difficile* Defined Medium (CDDM) (Karasawa et al., 1995) agar plates supplemented with FC (50 μg/ml) prior to patch-plating on BHIS agar plates supplemented with thiamphenicol to screen for plasmid excision. FC-resistant, thiamphenicol-sensitive clones were screened by PCR to separate mutants from WT revertants using primer pair 217–218. A detailed list of plasmids and primers used in this study is provided in Supplementary Tables S2 and S3, respectively.

### Construction of *sigEFG* Mutants

*sigEFG* mutants were created in the 630Δ*erm P_tet_-spo0A* background *via* allele-coupled exchange as described previously (Cartman et al., 2012). Briefly, the pMTL-SC7315 plasmid backbone was linearized by inverse PCR using primers 103 and 104. 900 bp upstream and downstream homology arms were PCR amplified from *C. difficile* 630 genomic DNA, using primer pairs 161–162 and 163–164 (*sigE*); 167–168 and 169–170 (*sigF*); 174–175 and 176–177 (*sigG*) respectively. The resulting fragments were ligated using Gibson assembly (NEB) as per manufacturer’s instructions yielding pMLD126 (*sigE*), pMLD127 (*sigF*), and pMLD128 (*sigG*). All plasmids were transformed into *E. coli* CA434 and conjugated into *C. difficile* 630Δ*erm P_tet_-spo0A* as described previously (Purdy et al., 2002). Resulting colonies were restreaked twice onto fresh BHIS agar plates supplemented with thiamphenicol and D-cycloserine to counter-select for *E. coli.* These were then restreaked onto CDMM agar plates supplemented with FC (50 μg/ml) before patch-plating on BHIS agar supplemented with thiamphenicol to screen for plasmid excision. FC-resistant, thiamphenicol-sensitive clones were screened by PCR to separate mutants from WT revertants using primer pairs 165–166 (*sigE*), 171–172 (*sigF*), and 177–178 (*sigG*).

### RNA Extraction and Quantitative Reverse Transcription PCR Analysis (qRT-PCR)

RNA was extracted from *C. difficile* cultures grown in BHIS to early stationary phase (OD_600_ ∼ 0.8) using the Total RNA Purification Kit (Norgen Biotek, Corp.) according to the manufacturer’s instructions. Purified RNA was DNase-treated using the Turbo DNA-free Kit (Life Technologies). First strand synthesis was performed on 1 μg of RNA using the SuperScript III VILO cDNA Synthesis Kit (Invitrogen) according to the manufacturer’s instructions. 50 ng of cDNA per reaction was used to perform qRT-PCR analysis using the SensiFast SYBR No-ROX Kit (Bioline) on a RotorGene 6000 instrument (Corbett Research). cDNA synthesis reactions containing no reverse transcriptase were included to control for genomic contamination. Primers for qRT-PCR analysis were designed using PrimerQuest (Integrated DNA Technologies), and primer efficiencies were calculated for each primer set prior to use. qRT-PCR was performed in technical triplicate for each cDNA sample and primer pair and on cDNA isolated from a minimum of two biological replicates. Results were calculated by the comparative cycle threshold method (Schmittgen and Livak, 2008) and normalized to the *rpoC* transcript. Results are presented as the means and standard deviations of the means and a two-tailed Student’s *t*-test was performed to determine statistical significance using GraphPad Prism 6.

### Sporulation Assays

*C. difficile* cultures were grown in BHIS broth to logarithmic growth phase (OD_600_ ∼ 0.6), diluted 1:10,000 in fresh broth, and grown overnight to stationary phase. This allowed synchronization of growth and minimized the carry-over of spores from initial cultures. Total and heat-resistant CFUs were then enumerated at 24 h intervals for 5 days. To this end, at each time point serial 10-fold dilutions were prepared in pre-reduced PBS and 20 μl were spotted in triplicate onto BHIS agar supplemented with 0.1% (w/v) sodium taurocholate. To determine the number of spores, samples were incubated at 70°C for 30 min before spotting onto plates. Colonies were enumerated after 24 h incubation in an anaerobic cabinet.

### Microscopy

0.5 ml samples from cultures grown in SM broth were harvested by centrifugation (2 min at 4,000 × *g*) 14 h after inoculation. Cells were washed with 1 ml of PBS, resuspended in 100 μl of PBS and spotted onto 1.5% (w/v) agarose pads supplemented with a lipophilic steryl membrane dye: *N*-(3-triethylammoniumprpyl)24-(*p*-diethylaminophenyl-hexatrienyl) pyridinium dibromide (FM4-64; Invitrogen; 1 μg/ml), a DNA dye: 2′-(4-hydroxyphenyl)-5-(4-methyl-1-piperazinyl)-2,5′-bi-1*H*-benzimidazole trihydrochloride hydrate, *bis*Benzimide (Hoechst 33258, Thermo Fisher; 1 μg/ml) and 0.5% (v/v) di-methylsulfoxide (DMSO). Images were captured using the Metamorph software package on a Nikon Ti microscope and were processed using ImageJ (Schindelin et al., 2012; Schneider et al., 2012).

### Western Blotting

To prepare *C. difficile* whole cell lysates, 0.5 ml samples from cultures grown overnight in BHIS to stationary phase were harvested by centrifugation (2 min at 4,000 × *g*), washed with PBS, resuspended to OD_600_ = 10 in sample buffer and boiled for 10 min. S-layer extracts were prepared using low pH glycine as previously described (Calabi et al., 2001). SDS-PAGE was carried out according to standard protocols. For immunoblot analysis, proteins were transferred to nitrocellulose membranes using the Trans-blot Turbo system (Bio-rad) according to the manufacturer’s instructions. Membranes were blocked in 3% (w/v) skimmed milk in PBS before probing with rabbit anti-Spo0A antibody at 1:5,000 or chicken anti-FliC at 1:100,000. Primary antibodies were detected using horseradish peroxidase (HRP)-conjugated goat anti-rabbit IgG (H+L) antibody at 1:2,500 (Promega) or goat anti-chicken IgY antibody at 1:10,000 (Novex, Life Technologies) and Clarity Western ECL substrate (Bio-rad).

### Animal Studies

12 C57BL/6 mice aged 6–7 weeks (Charles River) were pre-treated with drinking water containing 250 mg/ml clindamycin for 7 days. Clindamycin treatment was then removed for 24 h, after which the mice were infected by oral gavage with 1 × 10^7^ CFU P*_tet_*-*spo0A* grown for 16 h in TYG (3% w/v Bacto tryptose, 2% w/v yeast, 0.5% w/v glucose). The mice were split into two groups of 6, each receiving either 0.1 mg/ml ATc or 1% (v/v) ethanol in drinking water, refreshed every 24 h. Fresh feces were collected and suspended in sterilized distilled water to 0.2 g/ml. For heat-treated counts, the suspension was treated at 70°C for 30 min. 20 μl of suspension was plated in technical triplicate on Chrome ID plates (BioMerieux) and CFU/g fecal matter enumerated following 16 h growth.

## Ethics Statement

All animal work was performed under the United Kingdom Home Office project license PPL 70/8276 and with approval from the Royal Holloway University of London Ethics Committee.

## Author Contributions

MD and SW designed the study, developed the methodology, collected and analyzed the data, wrote and revised the manuscript; SH and HH collected the data; PS and SC designed the study, analyzed the data, revised the manuscript.

## Conflict of Interest Statement

The authors declare that the research was conducted in the absence of any commercial or financial relationships that could be construed as a potential conflict of interest.
