# Supplementary material for: Inducible Expression of spo0A as a Universal Tool for Studying Sporulation in Clostridium difficile
Source: Front Microbiol. 2017 Sep 21;8:1793. doi: 10.3389/fmicb.2017.01793 (PMC5613124; doi:10.3389/fmicb.2017.01793)
Supplement: Supplementary file 1 [file Data_Sheet_1.PDF]

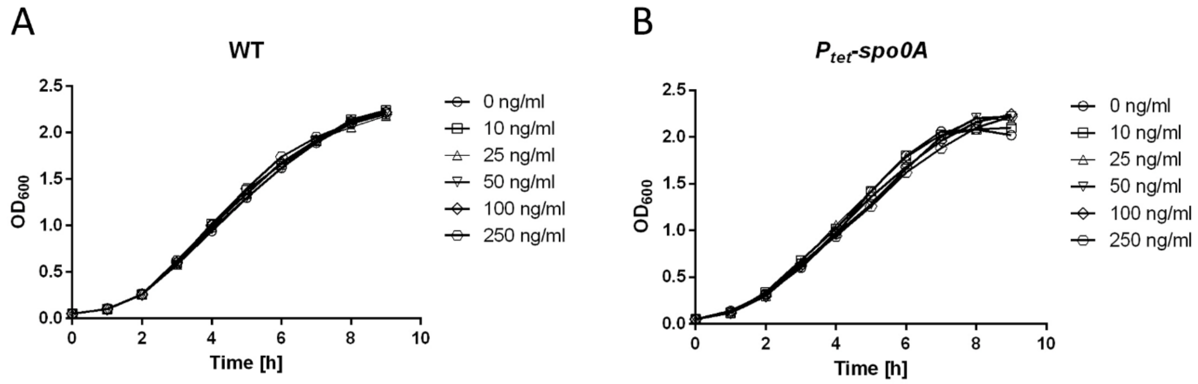

Figure S1. Growth curves of WT and P_{tet}*-spo0A* incubated with varying concentrations of ATc.

Overnight cultures of WT (A) or P_{tet}*-spo0A* (B) were sub-cultured to OD 0.05 in fresh medium containing the concentration of ATc indicated. OD_{600} measurements were taken every hour

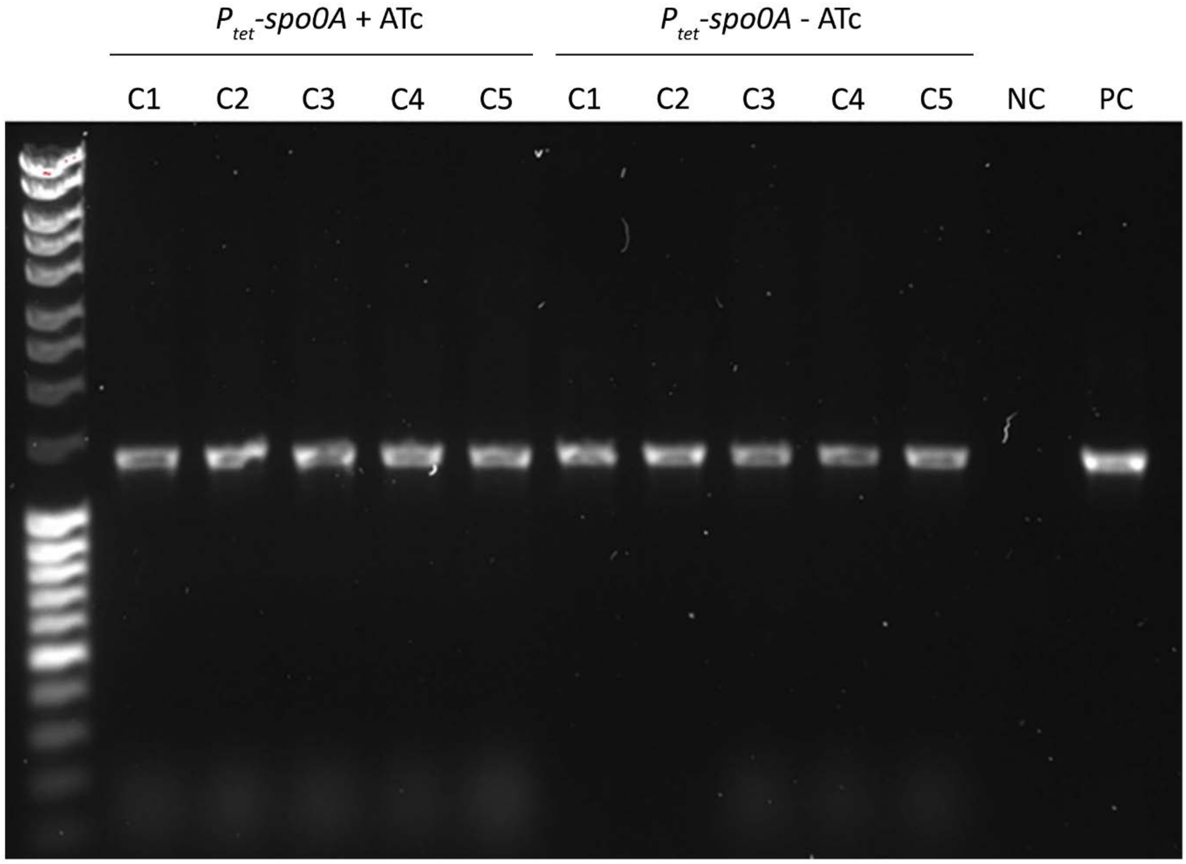

Figure S2. PCR screening of strains isolated from mouse feces.

To confirm the identiy of the strains isolated from mouse feces, 5 colonies from each group were picked at random. DNA was amplified using primers 217 and 219 which should yield a 2,624 bp product for the P_{tet}*-spo0A* strain and no product for the wildtype (see Figure 1 for diagram). NC - negative control (WT gDNA), PC - positive control (previously verified P_{tet}*-spo0A* gDNA).

Table S1. Strains used in this study

Strain	Source	Relevant details
E. coli		
CA434	Purdy *et al*., 2002	Conjugation donor strain. HB101-derivative carrying the conjugative plasmid R702
C. difficile		
630	Hussain *et al*., 2005	Virulent, multidrug-resistant epidemic strain isolated in Zurich in 1982. Ribotype 012; *tcdA+*; *tcdB+*
630Δ*erm*	Hussain *et al*., 2005	Erythromycin-sensitive derivative of *C. difficile* 630
630 *Ptet-spo0A*	This study	630 with *Ptet* inserted 30bp upstream of spo0A (CD1214) ORF obtained via *codA* ACE
630Δ*erm Ptet-spo0A*	This study	630Δ*erm* with *Ptet* inserted 30bp upstream of spo0A (CD1214) ORF obtained via *codA* ACE
630Δ*erm*Δ*sigE Ptet-spo0A*	This study	630Δ*erm Ptet-spo0A* carrying a 633 bp deletion (aa 11-222) in *sigE* (CD2643)
630Δ*erm*Δ*sigF Ptet-spo0A*	This study	630Δ*erm Ptet-spo0A* carrying a 705 bp deletion (aa 11-246) in *sigF* (CD0772)
630Δ*erm*Δ*sigG Ptet-spo0A*	This study	630Δ*erm Ptet-spo0A* carrying a 1,932 bp deletion (aa 11-655) in *sigG* (CD2642)

Table S2. Plasmids used in this study

Plasmid	Descriptive name	Source	Relevant details
pMTL-SC7315	pMTL8000-pCB102-catP-ColeE1+tra-T1-codA-T2	Cartman *et al.*, 2012	*E.coli – C. difficile* shuttle vector for *codA* ACE in *C. diffiicle* 630-derived strains
pSEW200	pMTL-SC7315-P_{tet}-*spo0A*	This study	Used to introduce the P_{tet} promoter 30bp upstream of spo0A (CD1214) ORF. 1,200 bp homology arms
pMLD126	pMTL-SC7315-Δ*sigE*	This study	Used to introduce a 633 bp deletion (aa 11-222) in *sigE* (CD2643) in *C. difficile* 630Δ*erm*. 900 bp homology arms
pMLD127	pMTL-SC7315-Δ*sigF*	This study	Used to introduce a 705 bp deletion (aa 11-246) in *sigF* (CD0722) in *C. difficile* 630Δ*erm*. 900 bp homology arms
pMLD128	pMTL-SC7315-Δ*sigG*	This study	Used to introduce a 1,932 bp deletion (aa 11-655) in *sigG* (CD2642) in *C. difficile* 630Δ*erm*. 900 bp homology arms

Table S3. Primers used in this study

Name	Sequence (5'-3')	Description
103	AAACTCCTTTTTGATAATCTCATGAC	pMTL-SC7315 linearisation primer
104	AAACTTAGGGTAACAAAAAACACC	pMTL-SC7315 linearisation primer
155	gtgtttttgttaccctaagtttTAATAATACAGCATAGGAGTAGATG	Gibson Assembly: Amplification of a 1,200 bp region upstream of *spo0A* for insertion into pMTL-SC7315
156	gcagaattcgAACTTTTTACACAATTGCTTCC	Gibson Assembly: Amplification of a 1,200 bp region upstream of *spo0A* for insertion into pMTL-SC7315
157	gtaaaaagttCGAATTCTGCATCAAGCTAG	Gibson Assembly: Amplification of P_{tet} from pRPF185 for insertion in between *spo0A* flanking arms
158	cagaaaactaGGAGCTCAGATCTGTTAAC	Gibson Assembly: Amplification of P_{tet} from pRPF185 for insertion in between *spo0A* flanking arms
159	tctgagctccTAGTTTTCTGTAATAAGAAGATGTTTTTTAATG	Gibson Assembly: Amplification of 5' of *spo0A* including RBS for insertion into pMTL-SC7315
160	agattatcaaaaaggagtttGTTTTAAGTTTGTTTTGCATCTTTG	Gibson Assembly: Amplification of 5' of *spo0A* including RBS for insertion into pMTL-SC7315
217	ATATTACCGCCCTTCAAAC	P_{tet}-*spo0A* screening
218	CTACTTTTATAGGGGCCTC	P_{tet}-*spo0A* screening
219	AAATGTGAAAGTGGGTC	P_{tet}-*spo0A* screening
220	AGCGCAATAAATCTAGGAGCA	*spo0A* (CD1214) qPCR primer
221	TGGCTCAACTTGTGTAACTCTAT	*spo0A* (CD1214) qPCR primer
222	TCAAGAGAGCTGCTGATGATG	*fliC* (CD0239) qPCR primer
223	CGTTTCTTCCTGCTTGGTCTA	*fliC* (CD0239) qPCR primer
230	GCAGGTATGCGTGGTCTTAT	*rpoC* (CD00670) qPCR primer
231	CTGGCTCCATGTGAAGATGT	*rpoC* (CD00670) qPCR primer
161	gtttttgttaccctaagtttAAAGCATTTTTTTCAATTCTTGATAC	Gibson Assembly: pMTL-SC7315 -> sigE LHR
162	gaataataagcTCTAGACTTGAAAAGAAAATAATCTC	Gibson Assembly: sigE LHR <- sigE RHR
163	aagtctagaGCTTATTATTCTTTCTTTCAATCG	Gibson Assembly: sigE LHR -> sigE RHR
164	gattatcaaaaaggagtttATGGTGTATATTGAGTACTATGTTATAG	Gibson Assembly: sigE RHR <- pMTL-SC7315
165	CCATGGATTACTTATACATATTTTCTC	*sigE* screening primer
166	CTATCTTATAATATGTTTAAAGGTTTTAAGG	*sigE* screening primer
167	gtttttgttaccctaagtttAATAAAAATATAATTATAGAATTGATTAAAAGTTACTATTTTTAC	Gibson Assembly: pMTL-SC7315 -> *sigF* LHR
168	cttagacaaTTTTTCTTCTCTGGCAACAG	Gibson Assembly: *sigF* LHR <- *sigF* RHR
169	agaagaaaaaTTGTCTAAGTTAAAAGAATATATATCGTG	Gibson Assembly: *sigF* LHR -> *sigF* RHR
170	gattatcaaaaaggagtttTTTAATTTTGCATTTTACTATTCAAATTTTTAATATTAATTAAAAATATTAATTAAATC	Gibson Assembly: *sigF* RHR <- pMTL-SC7315
171	GACAAAACTAGTTAAATTTCATTGAAGG	*sigF* screening primer
172	CAATTCTTTTATTTTTCATATTTTCACACC	*sigF* screening primer
173	gtttttgttaccctaagtttTATATTTATATAATCTTTCATCATTTGTCTC	Gibson Assembly: pMTL-SC7315 -> sigG LHR
174	atatgtggtGCTTTAAAAAATATGAGAAAATATGTATAAG	Gibson Assembly: sigG LHR <- sigG RHR
175	tttttaaagcACCACATATTTCAACCTTATTAAC	Gibson Assembly: sigG LHR -> sigG RHR
176	gattatcaaaaaggagtttTAATGAATCCAAATATATTGCCTG	Gibson Assembly: sigG RHR <- pMTL-SC7315
177	CTTAAATTAACATACTCTTTAATATCTTGTAC	*sigG* screening primer
178	CAAATTTTAATGATAGAGAATATGGAGC	*sigG* screening primer
